# *MfOfd1* is crucial for stress responses and virulence in the peach brown rot fungus *Monilinia fructicola*

**DOI:** 10.1111/mpp.12933

**Published:** 2020-04-21

**Authors:** Ming-Ming Zhang, Zuo-Qian Wang, Xiao Xu, Song Huang, Wei-Xiao Yin, Chao‐Xi Luo

**Affiliations:** ^1^ The Key Lab of Horticultural Plant Biology Ministry of Education Huazhong Agricultural University Wuhan China; ^2^ Institute of Plant Protection and Soil Fertilizer Hubei Academy of Agricultural Science Wuhan China; ^3^ Key Lab of Crop Disease Monitoring and Safety Control in Hubei Province and College of Plant Science and Technology Huazhong Agricultural University Wuhan China

**Keywords:** exogenous stresses, *MfOfd1* gene, *Monilinia fructicola*, redox response, sporulation, virulence

## Abstract

*Monilinia fructicola* is the most widely distributed species among the *Monilinia* genus in the world, and causes blossom blight, twig canker, and fruit rot on Rosaceae fruits. To date, studies on genomics and pathogenicity are limited in *M. fructicola*. In this study, we identified a redox‐related gene, *MfOfd1*, which was significantly up‐regulated at 1 hr after inoculation of *M. fructicola* on peach fruits. We used the clustered regulatory inter‐spaced short palindromic repeats (CRISPR)/Cas9 system combined with homologous recombination to determine the function of the *MfOfd1* gene. The results showed that the sporulation of knockdown transformants was reduced by 53% to 83%. The knockdown transformants showed increased sensitivity to H_2_O_2_ and decreased virulence on peach fruits compared to the wild‐type isolate Bmpc7. It was found that H_2_O_2_ could stimulate the expression of *MfOfd1* in the wild‐type isolate. The transformants were also more sensitive to exogenous osmotic stress, such as glycerol, d‐sorbitol, and NaCl, and to dicarboximide fungicides (iprodione and dimethachlon). These results indicate that the *MfOfd1* gene plays an important role in *M. fructicola* in sporulation, oxidative response, osmotic stress tolerance, and virulence.

## Graphical abstract

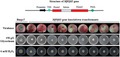

## 1 INTRODUCTION

*Monilinia fructicola* is an important Ascomycota pathogen in peach production that causes blossom blight, twig canker, and brown rot of fruits, with severe yield losses during both field production and post‐harvest processing (Luo, 2017). It not only infects stone fruits, for example peach, plum, and apricot (Ritchie, 2005; Hilber‐Bodmer *et al.*, 2010), but also damages apple, pear, and other pome fruits (Grabke *et al.*, 2011). Therefore, the occurrence of brown rot disease is one of the main factors that restrict the yield and quality of fruit production. In previous studies, some genes were found to be related to the redox state and played an important role in pathogenesis, for example an endopolygalacturonase (endo‐PG1)‐encoding gene was demonstrated to be crucial for pathogenicity in *M. fructicola* and *Botrytis cinerea* (Have *et al.*, 1998; Chou *et al.*, 2015). Compared with the wild‐type isolate, *MfPG1* overexpression transformants produced smaller lesions and higher levels of reactive oxygen species (ROS) on the petals of peach and rose flowers, suggesting that the decreased virulence in overexpression transformants might be caused by inducing ROS accumulation in the *Prunus–M. fructicola* interactions (Chou *et al.*, 2015). In addition, it was shown that cutinase MfCUT1 and activating protein MfAP1 are potent virulence determinants of *M. fructicola* (Lee *et al.*, 2010; Chiu *et al.*, 2013; Yu *et al.*, 2017). Furthermore, in the promoter of *MfCUT1*, several potential MfAP1‐binding sites were observed, suggesting that the expression of *MfCUT1* might be regulated by *MfAP1* (Yu *et al.*, 2017).

It has been reported that the failure of cells to respond to hypoxia may result in the death of cells and organisms (Gillies and Gatenby, 2007; Semenza, 2011). In *Schizosaccharomyces pombe*, the transcription factor Sre1 is a regulator of genes for adaptation to low oxygen conditions (Todd *et al.*, 2006). Under the hypoxic condition, the prolyl 4‐hydroxylase‐like 2‐OG‐Fe(Ⅱ)‐dependent dioxygenase (Ofd1) controls both DNA binding and degradation by regulation of the sterol regulatory element‐binding protein (SREBP) Sre1. Sre1 is proteolytically cleaved under low oxygen conditions, and its N‐terminal segment (Sre1N) serves as a hypoxic transcription factor, which enters the nucleus and up‐regulates genes essential for growth under low oxygen conditions. When oxygen is sufficient, the Ofd1 uses multiple domains to down‐regulate Sre1N activity by inhibiting Sre1N binding to DNA and accelerating Sre1N degradation. Ofd1 consists of two domains: an N‐terminal 2‐OG‐Fe(II) dioxygenase domain and a C‐terminal degradation domain (CTDD). The Ofd1 N‐terminal dioxygenase domain is required for oxygen sensing and regulating the ability of Ofd1 CTDD to destabilize Sre1N; its C‐terminal domain accelerates Sre1N degradation (Hughes and Espenshade, 2008; Lee *et al.*, 2014; Gu *et al* *.*, 2018). In the absence of oxygen, the N‐terminal dioxygenase domain inhibits the Ofd1 CTDD, leading to accumulation of Sre1N. It has also been reported that Nro1 (SPCC4B3.07) directly inhibits Ofd1 as a positive regulator of Sre1N stability. Under hypoxic conditions, Nro1 binds to Ofd1 CTDD and inhibits Sre1N degradation. In present of oxygen, the binding of Nro1 to Ofd1 is disrupted and leads to rapid degradation of Sre1N (Porter *et al.*, 2012; Yeh, 2012; Lee *et al.*, 2014). In this study, a homologous gene of the redox‐related *Ofd1* was significantly up‐regulated 1 hr after inoculation of *M. fructicola* (designated *MfOfd1*) on peach fruits, suggesting that it may play an important role in the pathogenicity of *M. fructicola*.

Oxygen (O_2_) is an indispensable substance in the process of cell life. Under anoxic or hypoxic conditions, the failure of cells to respond to hypoxia may lead to a series of diseases, and even cause the death of cells or organisms (Semenza, 2010, 2011). However, when O_2_ is oxidized in plants, it immediately produces toxic intermediate products (ROS), including superoxide radicals (O_2_^−^), hydroxyl radicals (^−^OH), singlet oxygen (^1^O_2_), and hydrogen peroxide (H_2_O_2_) (Mittler, 2017; Messens, 2018). ROS, as the by‐products of aerobic metabolism, can result in oxidation or damage to DNA, RNA, proteins, and membrane components (Beckman and Ames, 1998). As a class of signalling molecules, ROS play important roles in plant–pathogen interactions. On perception of pathogen attack, plants often produce a large amount of ROS, which regulate the redox state, signal transduction of stress, systemic resistance, and cell necrosis (Mittler *et al.*, 2004; O’Brien and Bolwell, 2012; Foyer and Noctor, 2013). In the ROS family, H_2_O_2_ plays a role as the main signalling molecule for plants to fight against pathogen invasion and improve plant resistance through inducing gene expression, activation of related enzymes, and programmed cell death (Pei *et al.*, 2000; Neill *et al.*, 2002; O’Brien and Bolwell, 2012).

The infection of biotrophic pathogens is extremely inhibited by ROS‐induced programmed cell death surrounding the infected host cells (Fath *et al.*, 2002), while necrotrophic pathogens, such as *Sclerotinia sclerotiorum* and *B. cinerea*, mainly secrete some enzymes, toxins, ROS, and other substances to kill the host cells and then obtain nutrients from the dead cells (Asai and Yoshioka, 2009; Rietz *et al.*, 2012). While the oxidative burst is important for their infection, necrotrophic pathogens can produce ROS by themselves or stimulate hosts to produce them (Kim *et al.*, 2008; Alkan *et al.*, 2009; Prusky *et al*., 2009). However, these pathogens need to activate the detoxification mechanism to respond to oxidative stress.

A few redox‐related genes required for cellular responses to oxidative/redox conditions have been studied in fungi. However, it is unclear whether and how ROS accumulation is involved in virulence in *M. fructicola* during pathogenesis. Previous studies showed that the cutinase‐encoding gene *MfCUT1* is a virulence factor. *MfCUT1* expression was up‐regulated by H_2_O_2_ and down‐regulated by antioxidants in axenic culture (Lee *et al.*, 2010; Chiu *et al.*, 2013). Meanwhile, an activating protein‐like transcription factor MfAP1 was identified in *M. fructicola*, which had several binding sites at the DNA sequence upstream of *MfCUT1* (Lee *et al* *.*, 2010). When *M. fructicola* infected fruits or flowers, the expression of *MfAP1* was activated and the genes responding to oxidative stress were up‐regulated at the infection site (Yu *et al.*, 2017). In some other plant‐pathogenic fungi, *MfAP1* homologues have also been identified, and all of them function in the redox stress response (Lev *et al.*, 2005; Lin *et al.*, 2009; Temme and Tudzynski, 2009; Guo *et al.*, 2011; Walther and Wendland, 2012; Montibus *et al.*, 2013). In *Alternaria alternata* and *Magnaporthe oryzae*, *AaAP1* and *MoAP1* also play roles in vegetative growth and pathogenicity (Yang *et al* *.*, 2009; Guo *et al* *.*, 2011).

In this study, the function of the *MfOfd1* gene was analysed through genetic transformation. It was found that knockdown of the *MfOfd1* gene led to pleiotropic phenotypes, including insufficient sporulation, decreased virulence, and sensitivity to stress. This study sheds some light on the function of the *MfOfd1* gene for virulence and ROS detoxification in *M. fructicola*, which could deepen our understanding of fungal Ofd1s.

## 2 RESULTS

### 2.1 The *MfOfd1* gene was up‐regulated in the early infection stage of *M. fructicola* on peach fruits

**FIGURE 1:**
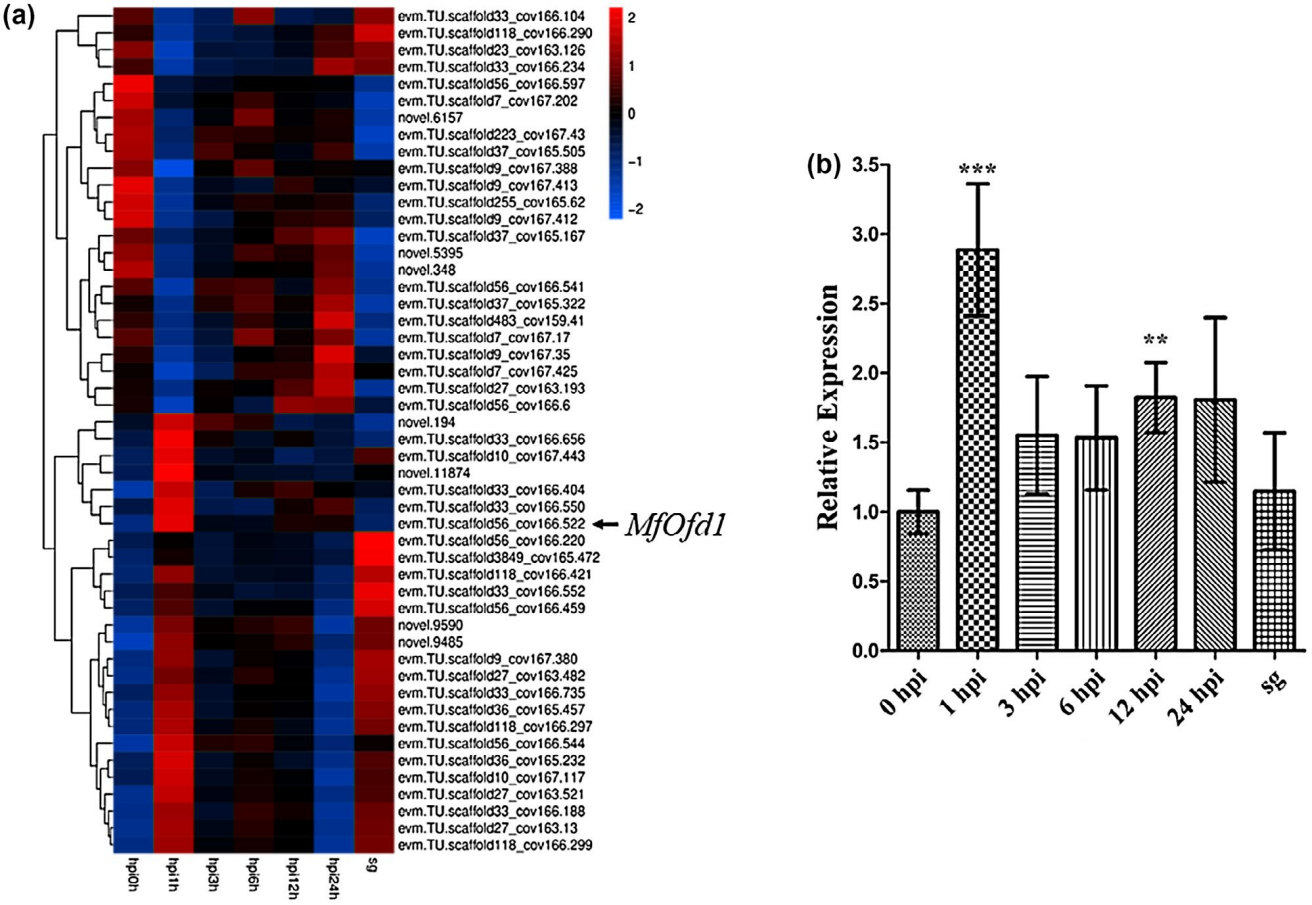
The expression level of *MfOfd1* in *Monilinia fructicola* during the early infection stage on peach fruits. (a) The heat map of partial differentially expressed genes at 1 hr post‐inoculation (hpi) is shown as an example. Red, blue, and black indicate the significantly up‐regulated, down‐regulated, and no difference genes, respectively. The selection criteria are |log_2_(fold change)| > 1 and *p*_adj_ < .05. (b) Relative expression of *MfOfd1* was confirmed by quantitative reverse transcription PCR at 0, 1, 3, 6, 12, and 24 hpi, and spore germination (sg). Significance was determined by *t* test (***p < .001, **p < .01)

*M. fructicola* isolate Bmpc7 was inoculated onto peach fruits, the different infection stage transcripts were examined by RNA‐Seq, and the transcription data were deposited in GenBank (accessions SAMN12871599 to SAMN12871619). Compared with the expression level at 0 hr post‐inoculation (hpi), a total of 188 differentially expressed genes were detected at 1 hpi, of which 100 genes were up‐regulated and 88 genes were down‐regulated (partial data are shown in Figure 1a). This early stage (1 hpi) was an important period for the peach–*M. fructicola* interaction; previous studies showed that some pathogenicity‐related genes (*MfCUT1* and *MfPGs* in *M. fructicola*, *SsSSVP1* in *S. sclerotiorium*) were up‐regulated at early stages of infection (Lee *et al* *.*, 2010; Chou *et al.*, 2015; Lyu *et al.*, 2016; Yu *et al* *.*, 2017). Based on the transcriptomic analysis and quantitative reverse transcription PCR (RT‐qPCR) analysis, the *MfOfd1* gene (MN515052) was up‐regulated during different infection stages, especially at 1 hpi (Figure 1b).

### 2.2 Characterization of *MfOfd1* from *M. fructicola*

**FIGURE 2:**
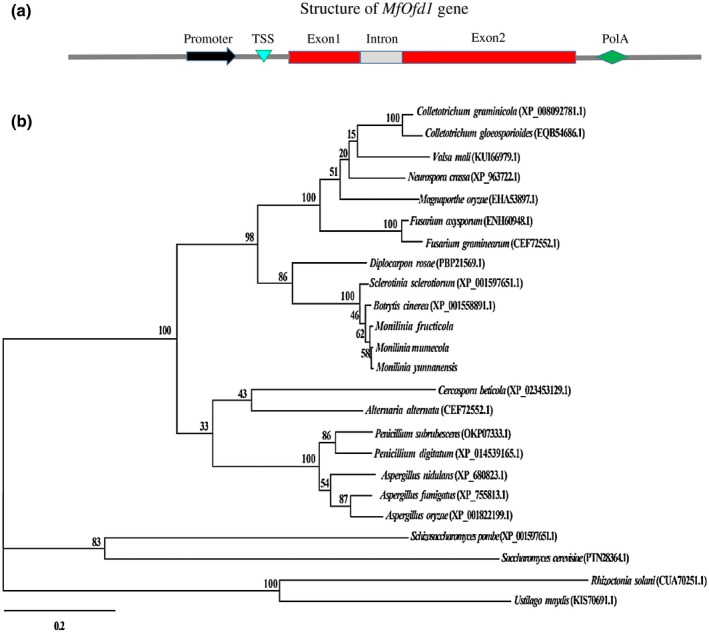
Bioinformatic analysis of Ofd1 protein. (a) The structure of the *Ofd1* gene (*MfOfd1*) in *Monilinia fructicola*. TSS, transcriptional start site; PolA, poly(A) tail. Not drawn to scale. (b) Phylogenetic analysis of Ofd1 homologues from 24 fungal species. The phylogenetic tree (maximum‐likelihood method) was constructed using MEGA v. 7 software

The *MfOfd1* genomic DNA and complementary DNA (cDNA) sequences were obtained using primer pair Ofd1‐For and Ofd1‐Rev with the genomic DNA and cDNA of the isolate Bmpc 7 as templates. Alignment of the genomic DNA sequence with the cDNA sequence revealed that the *MfOfd1* gene contains a 2,037 bp coding sequence (CDS) with a 61 bp intron (Figure 2a). The *MfOfd1* gene was predicted to encode a protein with 678 amino acids. The homologous proteins of MfOfd1 were identified by protein Blast and Pfam domain analysis. The results showed that Ofd1s are widely conserved in ascomycetes. The MfOfd1 amino acid sequence showed 83.57% and 83.25% identity with the Ofd1 homologues in *B. cinerea* (XP_001558891.1) and *S. sclerotiorum* (XP_001597651.1) (Figures 2b and S1). The domain analysis showed that MfOfd1 possesses a 2‐OG‐Fe(II) oxygenase superfamily domain and an oxoglutarate and iron‐dependent oxygenase degradation domain at the C‐terminus, which does not contain transmembrane domain.

### 2.3 Generation and characterization of *MfOfd1* knockdown transformants

**FIGURE 3:**
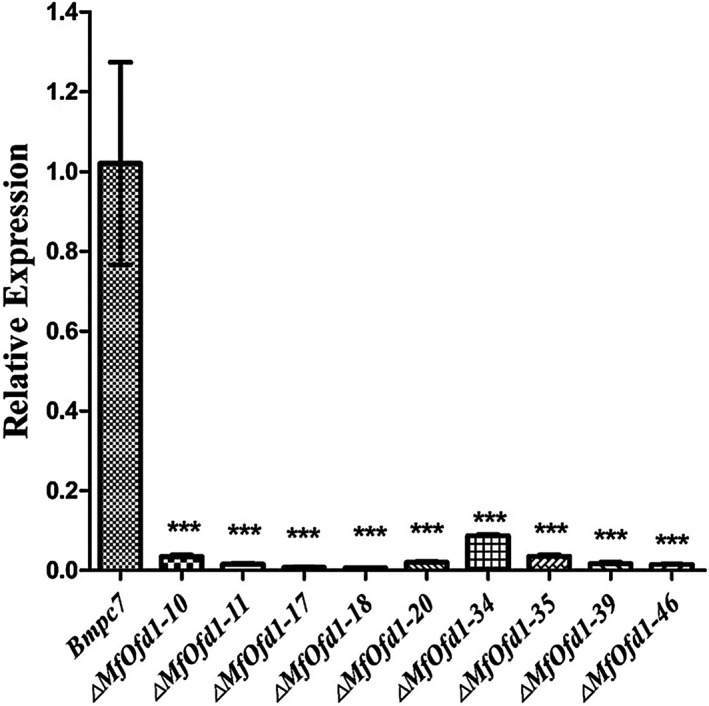
Relative expression of the *MfOfd1* gene in Bmpc7 and Δ*MfOfd1* transformants. The wild‐type isolate Bmpc7 and Δ*MfOfd1* transformants were cultured in potato dextrose broth for 36 hr, then the expression of *MfOfd1* was evaluated by quantitative reverse transcription PCR and the *β*‐*tubulin* gene was used as internal reference gene. The experiment was repeated three times independently. The processed data were analysed and plotted by GraphPad Prism v. 5.0 software. Significance was determined by *t* test (***p < .001)

To investigate the biological function of *MfOfd1* in *M. fructicola*, we tried to knock out the *MfOfd1* gene by homologous recombination and the CRISPR/Cas9 system, via polyethylene glycol (PEG)‐mediated protoplast transformation (Figures S2 and S3). The transformants were confirmed by PCR verification and RT‐qPCR (Figures S2 and S4). It should be noted that the primer pair MfOfd1‐C/Z‐For and MfOfd1‐C/Z‐Rev amplified two fragments of 876 and 1,829 bp from all of the obtained transformants, while the primers only amplified a single fragment of 876 bp from the wild‐type isolate, indicating that the transformants were heterokaryons (Figure S4). Therefore, the transformants were called "knockdown" transformants instead of "knockout" transformants. We obtained nine positive knockdown transformants: Δ*MfOfd1‐10*, Δ*MfOfd1‐11*, Δ*MfOfd1‐17*, Δ*MfOfd1‐18*, Δ*MfOfd1‐20*, Δ*MfOfd1‐34*, Δ*MfOfd1‐35*, Δ*MfOfd1‐39*, and Δ*MfOfd1‐46*. Nevertheless, the *MfOfd1* expression level in these knockdown transformants were reduced by 82.1%–98.2%, significantly lower than that in the wild‐type isolate (Figure 3).

### 2.4 Knockdown of *MfOfd1* influenced the sporulation of *M. fructicola*

**FIGURE 4:**
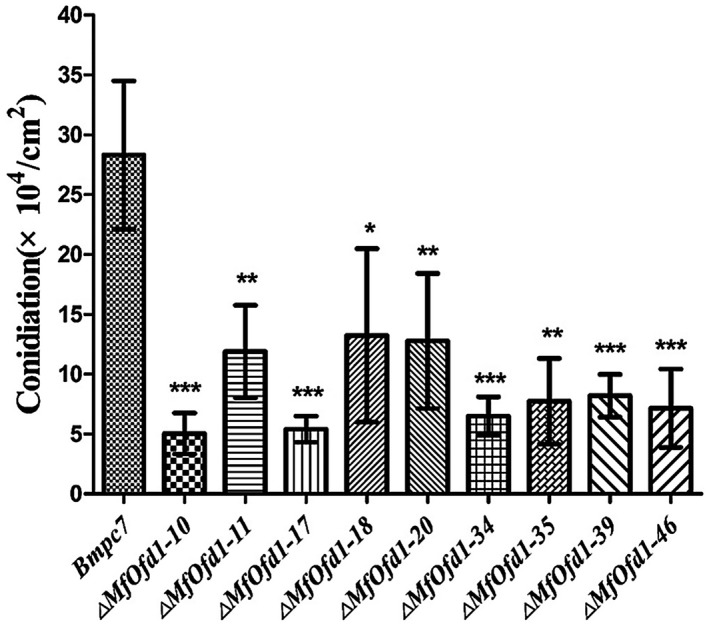
Conidiation of the wild‐type isolate Bmpc7 and Δ*MfOfd1* transformants. Conidia were collected from 20% V8 agar after incubation for 14 days. Significance was determined by *t* test (***p < .001, **p < .01, *p < .05)

In general, the phenotypes of transformants were similar. All of the transformants did not show differences in colony morphology, mycelial growth on potato dextrose agar (PDA), and minimal medium agar (MMA) (Table 1). As for the sporulation, transformants produced 0.5–1.32 × 10^5^ conidia/cm^2^, which was significantly lower than that of the wild‐type (2.83 × 10^5^ conidia/cm^2^) on V8 agar (Figure 4). To further elucidate how the defect of sporulation occurred in the aforementioned transformants, the conidiophores, conidial size, and conidial germination in transformants were compared with those of the parental wild‐type isolate Bmpc7. The results showed that the conidiophore structures and conidial germination of the transformants were normal and similar to that of the wild‐type isolate, while the conidial width of the transformants was slightly narrower than that observed in the wild‐type isolate (Table 1).

**TABLE 1:** Mycelial growth rate, sporulation, spore size, and germination of *MfOfd1* knockdown transformants

Strain	Mycelial growth rate (mm/day)		Sporulation (×10^5^/cm^2^)	Conidial size (um)		Conidial germination (%)
	PDA	MMA		Average (L × W)	Range (L × W)	
Bmpc7	16.61 ± 0.35a	14.61 ± 0.10ab	2.83 ± 0.62a	13.47 × 9.18	6.5‐17.8 × 5.1‐14.6	88.37 ± 0.03ab
Δ*MfOfd1‐10*	16.44 ± 0.42a	14.39 ± 0.25ab	0.50 ± 0.17d	12.30 × 8.43	8.3‐17.6 × 5.2‐11.1	90.06 ± 0.03ab
Δ*MfOfd1‐11*	16.33 ± 0.17a	14.17 ± 0.29a	1.19 ± 0.39bc	13.35 × 8.81	9.6‐17.8 × 6.2‐12.3	89.11 ± 0.02ab
Δ*MfOfd1‐17*	16.44 ± 0.25a	14.78 ± 0.19ab	0.54 ± 0.11cd	13.97 × 8.99	11.3‐18.9 × 6.5‐11.0	90.86 ± 0.02b
Δ*MfOfd1‐18*	16.33 ± 0.44a	15.22 ± 0.63b	1.32 ± 0.73b	13.60 × 8.34	9.2‐17.8 × 5.9‐10.7	86.50 ± 0.05a
Δ*MfOfd1‐20*	16.50 ± 0.33a	14.83 ± 0.17ab	1.28 ± 0.56b	14.40 × 8.17	9.8‐18.8 × 3.6‐11.8	86.33 ± 0.02a
Δ*MfOfd1‐34*	16.44 ± 0.48a	14.44 ± 0.54ab	0.65 ± 0.16bcd	13.92 × 7.95	11.5‐16.8 × 5.5‐10.9	86.33 ± 0.04a
Δ*MfOfd1‐35*	16.50 ± 0.33a	14.50 ± 0.44ab	0.77 ± 0.36bcd	14.34 × 8.26	10.4‐19.2 × 5.3‐11.8	86.09 ± 0.00a
Δ*MfOfd1‐39*	16.34 ± 0.17a	14.94 ± 0.19ab	0.82 ± 0.18bcd	13.76 × 7.83	9.2‐18.3 × 5.4‐10.9	88.67 ± 0.05ab
Δ*MfOfd1‐46*	16.45 ± 0.42a	14.06 ± 0.38a	0.72 ± 0.33bcd	13.21 × 8.58	9.5‐17.4 × 6.2‐12.1	90.08 ± 0.06ab

*Note.* Mean ± *SD*; values within the same column followed by the same letters are not significantly different based on one‐way analysis of variance with the LSD test in SPSS 21.0 software at *p = *.05. L, conidial length; MMA, minimal medium agar; PDA, potato dextrose agar; W, conidial width.

### 2.5 Knockdown of *MfOfd1* influenced the virulence of *M. fructicola* on peach fruits

**FIGURE 5:**
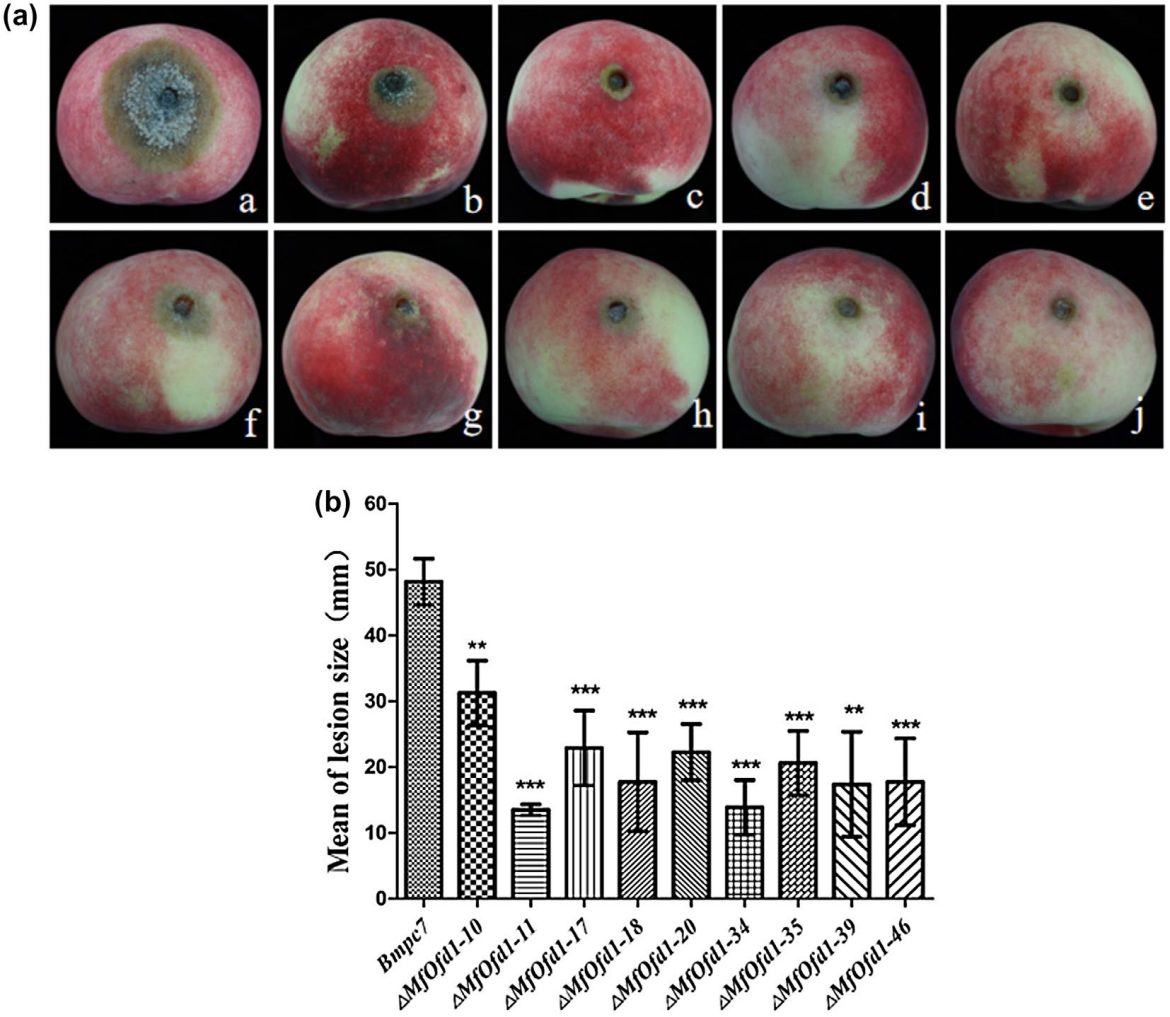
Virulence of wild‐type isolate Bmpc7 and *ΔMfOfd1* transformants. (a) Mycelial plugs of Bmpc7 and transformants were inoculated on peach fruits and incubated at 22 °C for 2 days. Strains from left to right are Bmpc7, Δ*MfOfd1‐10*, Δ*MfOfd1‐11*, Δ*MfOfd1‐17*, Δ*MfOfd1‐18*, Δ*MfOfd1‐20*, Δ*MfOfd1‐34*, Δ*MfOfd1‐35*, Δ*MfOfd1‐39*, and Δ*MfOfd1‐46*. (b) Lesions were measured at 2 days post‐inoculation. GraphPad Prism v. 5.0 software was used to analyse the mean and *SD*. Significance was determined by *t* test (***p < .001, **p < .01). Three independent biological replications were conducted

In order to know whether *MfOfd1* affects the virulence of *M. fructicola*, a virulence assay was performed on detached peach fruits. The results showed that the wild‐type isolate produced large and oval lesions on which there were dense grey hyphae and lots of spores, leading to typical peach brown rot disease symptoms, but the transformants produced smaller lesions with sparse hyphae, or even no visible hyphae on some lesions (Figure 5a). The lesions produced by the wild‐type isolate expanded rapidly with an average growth rate of 21.5 mm/day, while lesion expansion of transformants on the surface of fruits was slow, with an average growth rate of 4.3–12.5 mm/day (Figure 5b). In addition, the hyphae of transformants were significantly limited to the inoculation sites. However, as time progressed, the virulence difference between the wild‐type isolate and the transformants gradually decreased. These results indicate that the *MfOfd1* gene is important for the virulence of *M. fructicola*, especially in the early stage of infection.

### 2.6 *MfOfd1* is involved in the regulation of response to oxidative stress

**FIGURE 6:**
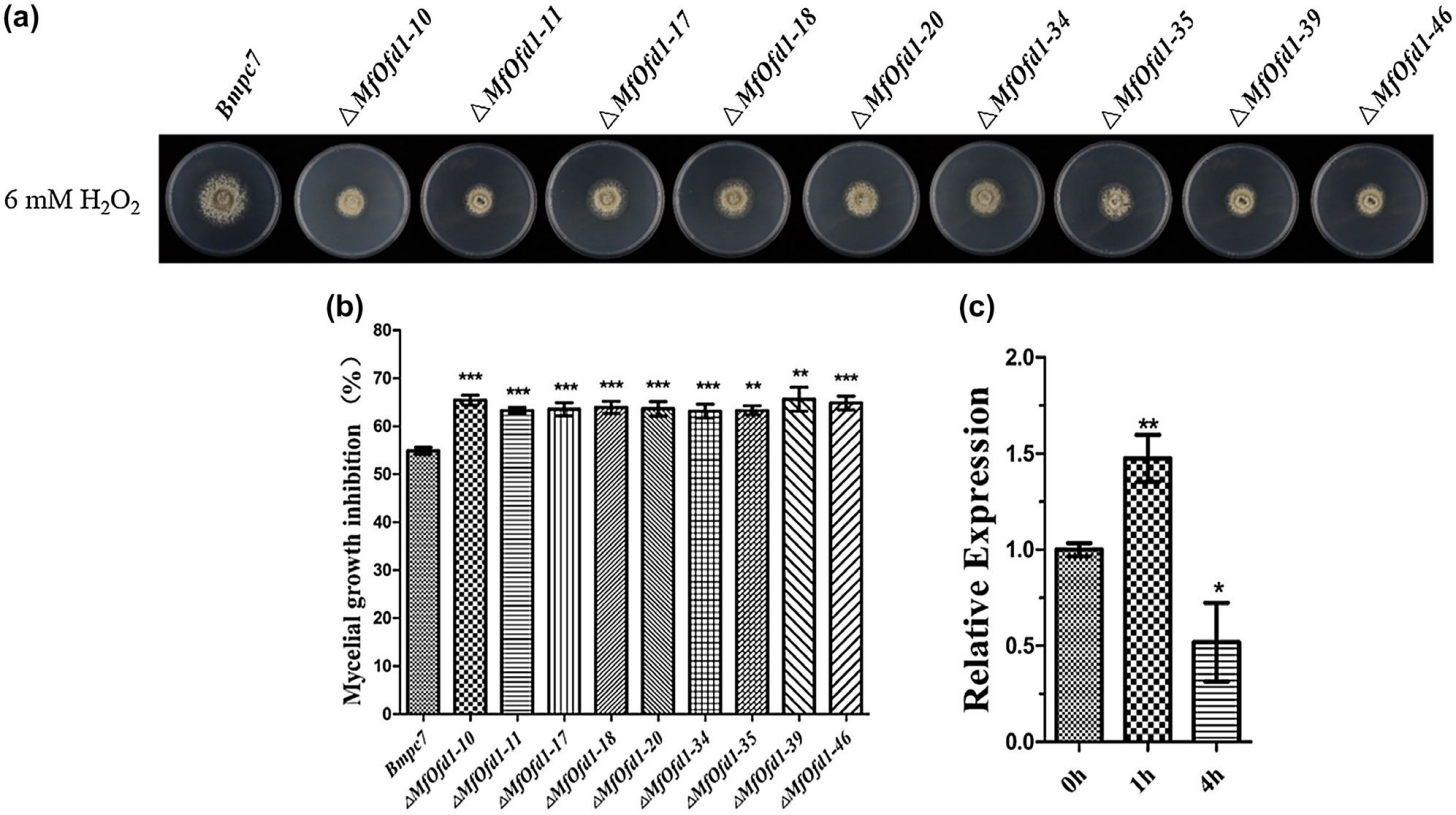
Mycelial growth under oxidative stress. (a) Colony growth of the wild‐type isolate Bmpc7 and Δ*MfOfd1* transformants in the presence of H_2_O_2_ (6 mM). (b) The inhibition rate of mycelial growth in Bmpc7 and Δ*MfOfd1* transformants on potato dextrose agar containing 6 mM H_2_O_2_. (c) The wild‐type isolate Bmpc7 was cultured in potato dextrose broth for 36 hr, then treated with 6 mM H_2_O_2_ for 0, 1, and 4 hr. The expression level of *MfOfd1* was detected by quantitative reverse transcription PCR. Significance was determined by *t* test (***p < .001, **p < .01, *p < .05)

ROS play an important role in plant defence. To elucidate the roles of *MfOfd1* to oxidative stress in *M. fructicola*, mycelial growth on PDA amended with H_2_O_2_ was measured for the wild‐type isolate and knockdown transformants. In the plates containing 6 mM H_2_O_2_, the inhibition of the wild‐type isolate was 54.8% and that of knockdown transformants was 63.2%–65.6% more sensitive to H_2_O_2_ (Figure 6a,b; Table 2). To further confirm that *MfOfd1* is involved in the oxidative stress response, mycelia of the wild‐type isolate cultured in potato dextrose broth (PDB) for 36 hr were treated with 6 mM H_2_O_2_, then the expression of *MfOfd1* was detected by RT‐qPCR. The result showed that compared with untreated mycelia (0 hr), the expression level of *MfOfd1* significantly up‐regulated at 1 hr of treatment, while the expression of *MfOfd1* decreased after 4 hr of treatment (Figure 6c). This result also indicates that *MfOfd1* is involved in the regulation of the oxidative stress response.

**TABLE 2:** Sensitivity of Bmpc7 and Δ*MfOfd1* strains to glycerol, d‐sorbitol, NaCl, H_2_O_2_, sodium dodecyl sulphate (SDS), Congo Red, and MgSO_4_

Strain	Mycelial growth inhibition (%)						
	150 g/L glycerol	1.2 M d‐sorbitol	0.6 M NaCl	6 mM H_2_O_2_	0.01% SDS	600 μg/ml Congo Red	0.7 M MgSO_4_
Bmpc7	33.78 ± 0.02b	34.45 ± 0.01b	46.18 ± 0.02e	54.85 ± 0.01b	56.25 ± 0.91bc	24.99 ± 1.96a	31.52 ± 4.37ab
Δ*MfOfd1‐10*	44.26 ± 0.02a	45.27 ± 0.00a	58.42 ± 0.01d	65.54 ± 0.01a	60.48 ± 0.43a	26.08 ± 2.64a	35.17 ± 2.61a
Δ*MfOfd1‐11*	43.54 ± 0.03a	45.28 ± 0.01a	63.10 ± 0.02c	63.27 ± 0.01a	58.25 ± 3.49ab	27.57 ± 1.77a	31.44 ± 2.14ab
Δ*MfOfd1‐17*	43.24 ± 0.02a	45.95 ± 0.02a	66.32 ± 0.01abc	63.51 ± 0.01a	58.57 ± 1.45ab	25.58 ± 2.64a	27.23 ± 2.07b
Δ*MfOfd1‐18*	44.56 ± 0.03a	44.73 ± 0.01a	70.14 ± 0.02a	63.95 ± 0.01a	59.31 ± 2.58ab	26.68 ± 2.53a	33.75 ± 3.51ab
Δ*MfOfd1‐20*	44.78 ± 0.02a	45.62 ± 0.02a	55.63 ± 0.03d	63.64 ± 0.02a	57.39 ± 0.53abc	26.07 ± 3.85a	28.05 ± 3.15ab
Δ*MfOfd1‐34*	43.58 ± 0.02a	44.43 ± 0.03a	68.66 ± 0.01ab	63.18 ± 0.02a	56.69 ± 0.27abc	25.70 ± 1.07a	31.76 ± 2.27ab
Δ*MfOfd1‐35*	44.44 ± 0.01a	46.46 ± 0.02a	68.35 ± 0.02ab	63.30 ± 0.02a	53.53 ± 0.47c	25.58 ± 0.96a	29.37 ± 1.90ab
Δ*MfOfd1‐39*	44.56 ± 0.01a	46.60 ± 0.01a	65.32 ± 0.05bc	65.65 ± 0.03a	59.26 ± 0.71ab	23.22 ± 2.49a	31.63 ± 2.60ab
Δ*MfOfd1‐46*	45.95 ± 0.02a	47.30 ± 0.01a	67.49 ± 0.02ab	64.86 ± 0.01a	59.00 ± 0.97ab	26.14 ± 1.99a	34.81 ± 2.41a

*Note*. Mean ± *SD*; values within the same column followed by the same letters are not significantly different based on one‐way analysis of variance with the LSD test in SPSS 21.0 software at *p = *.05.

### 2.7 *MfOfd1* is important for stress tolerance

**FIGURE 7:**
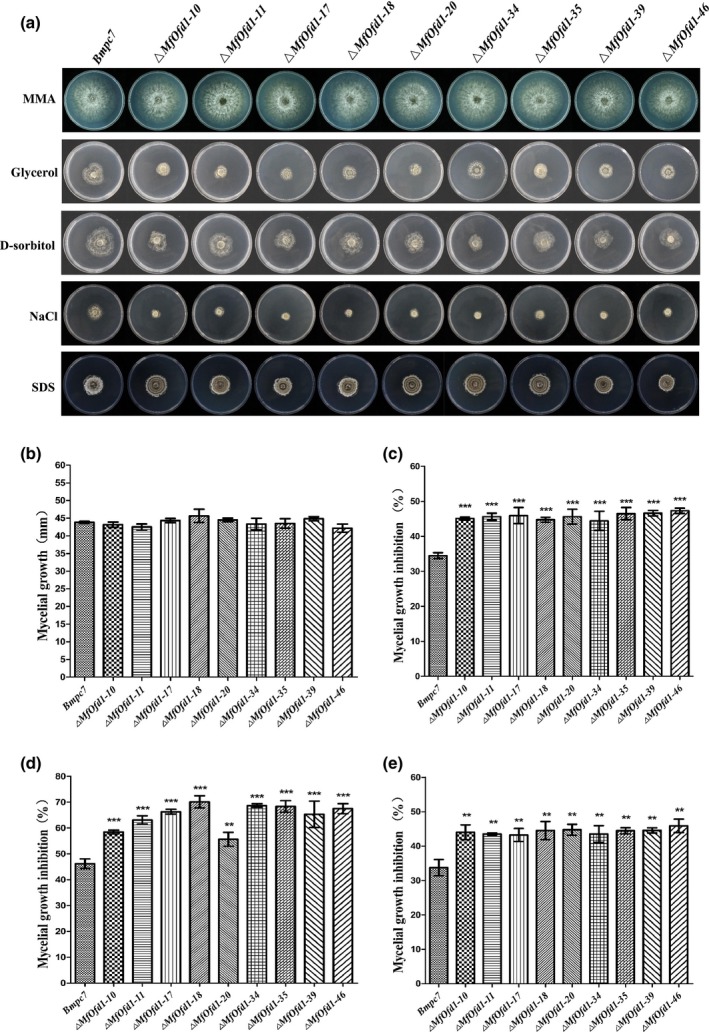
Mycelial growth under different stress conditions. (a) Comparison of the sensitivities of the parental wild‐type isolate Bmpc7 and Δ*MfOfd1* transformants to stresses mediated by minimal medium agar (MMA), glycerol (150 g/L),  d‐sorbitol (1.2 M), NaCl (0.6 M), and sodium dodecyl sulphate (SDS) (0.01%). (b) Nutrient starvation stress on MMA. Osmotic stress on potato dextrose agar containing 1.2 M d‐sorbitol (c), 0.6 M NaCl (d), and 150 g/L glycerol (e). Significance was determined by *t* test (***p < .001, **p < .01)

In *S. pombe*, Ofd1 is involved in regulating the Sre1N and sterol regulatory elements (SRE); sterol is widely present in biological cells and tissues, with different biological functions (Hughes and Espenshade, 2008; Yeh, 2012; Lee *et al.*, 2014). To evaluate whether *MfOfd1* is involved in regulating *M. fructicola* to exogenous stress responses, the mycelial inhibition ratio was measured for different stresses: 150 g/L glycerol, 1.2 M d‐sorbitol, 0.6 M NaCl, 0.01% sodium dodecyl sulphate (SDS), 600 μg/ml Congo Red, and 0.7 M MgSO_4_. The results showed that knockdown transformants were more sensitive to glycerol, sorbitol, and NaCl compared to the wild‐type isolate (Figure 7 and Table 2). No significant difference was observed for sensitivity to Congo Red and MgSO_4_ between transformants and the wild‐type isolate (Table 2). Under SDS stress, the colony of the wild type was fluffy and the mycelia were off‐white, while the colonies of transformants were dense and dark brown, and produced large amounts of conidia, which showed concentric sporodochia (Figure 7a). These results indicate that the deletion of *MfOfd1* did not affect the integrity of the *M. fructicola* cell wall, but may have an impact on the membrane protein.

### 2.8 *MfOfd1* influenced the sensitivities of *M. fructicola* to fungicides

**FIGURE 8:**
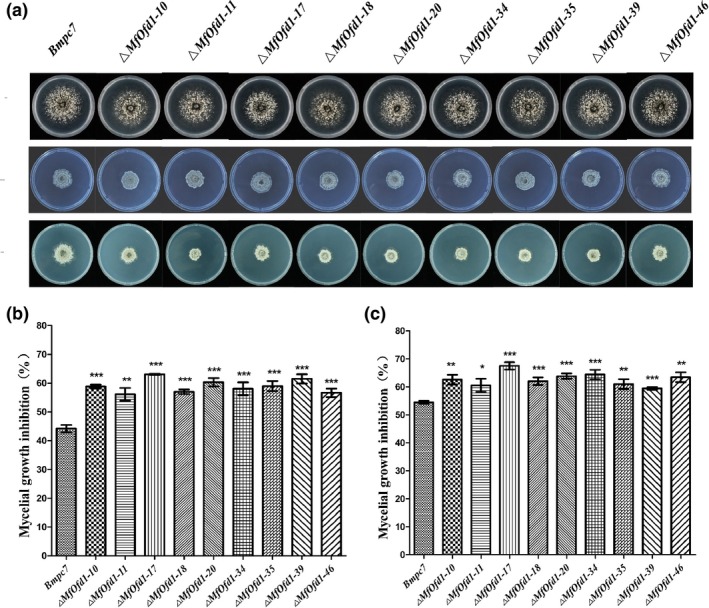
The Δ*MfOfd1* transformants showed increased sensitivity to dicarboximide fungicides (DCFs). (a) Colony growth of the wild‐type isolate Bmpc7 and Δ*MfOfd1* transformants on potato dextrose agar amended with the demethylation inhibitor fungicide propiconazole (0.2 μg/ml) or the DCF iprodione (0.2 μg/ml). Mycelial growth inhibition with the DCFs iprodione (b; 0.2 μg/ml) or dimethachlon (c; 0.5 μg/ml). Mycelial plugs (5 mm) of the Bmpc7 and Δ*MfOfd1* transformants were placed in the centre of those media and incubated at 22 °C for 3 days. Significance was determined by *t* test (***p < .001, **p < .01, *p < .05)

Application of fungicides is the most effective way to prevent and cure peach brown rot. In order to evaluate whether the deletion of *MfOfd1* affects the sensitivity of *M. fructicola* to generally applied fungicides, the mycelial inhibition ratio was measured on PDA amended with different types of fungicides, for example the dicarboximide fungicides (DCFs) iprodione and dimethachlon, the demethylation inhibitor fungicides (DMIs) propiconazole and tebuconazole, and the succinate dehydrogenase inhibitor fungicide (SDHI) boscalid. Under treatment with the DCFs iprodione or dimethachlon, the knockdown transformants showed increased sensitivity compared to the wild‐type isolate (Figure 8 and Table 3). Because the target of DCFs is the osmotic stress signal transduction pathway (Motoyama *et al.*, 2005), this result further supported the fact that *MfOfd1* plays an important role in the osmotic stress signal transduction pathway in *M. fructicola*. Under treatment with DMIs and SDHIs, the sensitivity did not show a significant difference between transformants and the parental wild‐type isolate (Table 3).

**TABLE 3:** Sensitivity of Bmpc7 and Δ*MfOfd1* strains to five fungicides

Strains	Mycelial growth inhibition (%)				
	0.2 μg/ml iprodione	0.5 μg/ml dimethachlon	0.2 μg/ml propiconazole	0.2 μg/ml tebuconazole	0.8 μg/ml boscalid
*Bmpc*7	44.22 ± 1.28e	54.46 ± 0.54d	56.10 ± 0.71ab	51.50 ± 0.81b	47.16 ± 2.96abc
Δ*MfOfd1‐10*	58.64 ± 0.67bcd	62.59 ± 1.72bc	56.78 ± 2.72ab	52.43 ± 2.40ab	47.12 ± 1.47abc
Δ*MfOfd1‐11*	56.11 ± 2.18d	60.55 ± 2.36bc	58.16 ± 1.28ab	52.84 ± 0.27ab	42.72 ± 2.18c
Δ*MfOfd1‐17*	63.06 ± 0.18a	67.51 ± 1.33a	57.63 ± 1.16ab	53.25 ± 0.03ab	49.61 ± 1.30a
Δ*MfOfd1‐18*	56.97 ± 0.82cd	62.04 ± 1.36bc	57.91 ± 1.16ab	52.87 ± 0.98ab	43.80 ± 2.04bc
Δ*MfOfd1‐20*	60.32 ± 1.45abc	63.81 ± 0.95b	58.10 ± 1.65ab	52.15 ± 1.43ab	49.27 ± 2.66ab
Δ*MfOfd1‐34*	58.06 ± 2.22bcd	64.42 ± 1.70ab	55.03 ± 1.31b	53.06 ± 1.88ab	46.44 ± 4.83abc
Δ*MfOfd1‐35*	58.98 ± 1.74bcd	61.01 ± 1.73bc	55.93 ± 0.33ab	53.75 ± 0.04ab	46.00 ± 1.62abc
Δ*MfOfd1‐39*	61.49 ± 1.59ab	59.46 ± 0.47c	56.09 ± 1.07ab	53.99 ± 0.25a	44.96 ± 4.71abc
Δ*MfOfd1‐46*	56.65 ± 1.48cd	63.46 ± 1.75b	58.70 ± 0.44a	53.75 ± 0.55ab	45.01 ± 3.08abc

*Note*. Mean ± *SD*; values within the same column followed by the same letters are not significantly different based on one‐way analysis of variance with the least‐significant difference (LSD) test in SPSS 21.0 software at *p = *.05.

## 3 DISCUSSION

The CRISPR/Cas9 system was originally discovered in bacteria and archaea as a defence system against phage and plasmids (Barrangou *et al.*, 2007). The CRISPR/Cas9 system has the advantages of convenient transformation, ease of vector construction, and high efficiency. Recently, the system has been successfully modified for genome editing in filamentous fungi (Arazoe *et al.*, 2015; Katayama *et al.*, 2016; Schuster *et al.*, 2016). As *M. fructicola* mycelia and conidia are multinucleate, either *Agrobacterium* T‐DNA mediated transformation or PEG‐mediated protoplast transformation may contain mixed nuclei with different integration patterns (Chen *et al.*, 2016). In this study, we combined homologous recombination with CRISPR/Cas9 to knock down the *MfOfd1* gene and obtained more transformants than previously using PEG‐mediated transformation (data not shown). However, PCR detection revealed that all these transformants were heterozygous. Nevertheless, the RT‐qPCR results showed that the expression level of *MfOfd1* in all of the knockdown transformants decreased significantly. Thus, these transformants could be used for further investigation of different phenotypes. As the knockdown transformants were heterozygotes containing both knockout and wild‐type genotypes, it is not necessary to complement the *MfOfd1.* However, further knockdown transformants may be needed to evaluate different phenotypes and obtain more reliable results.

Even though *MfOfd1* is not essential for mycelial growth in *M. fructicola*, it is important for sporulation. Conidiation is a key factor in the epidemics of peach brown rot (Ritchie, 2005), but reports about conidiation‐related genes are limited in *M. fructicola*. In this study, we found that the conidiation of knockdown transformants was reduced remarkably in comparison to the wild‐type isolate. It is possible that the knockdown of *MfOfd1* affects the expression of conidiation‐related genes, resulting in the decline of conidiation. These results indicate that *MfOfd1* is crucial for conidiation in *M. fructicola*. As far as we know, this is the first time a gene associated with conidiation in *M. fructicola* has been found*.*

Because *MfOfd1* was significantly up‐regulated at the early stages of infection, especially at 1 hpi, it was thought to contribute to the virulence. As shown in the results, the virulence assay confirmed the speculation that the *MfOfd1* gene is important for virulence. It is reasonable that *MfOfd1* plays an important role in tolerance to oxidative stress produced by the plant. Tolerance to osmotic and oxidative stress is important for infection by fungi (Lim *et al.*, 2018). In *M. fructicola*, the sterol regulatory element‐binding protein MfSre1 is associated with osmotic tolerance and resistance to DCFs, that is, the sensitivities to metal ions (NaCl, KCl, MgSO_4_), saccharide compounds (glucose, sucrose), cell wall and cell membrane damage agents (SDS, Congo Red) were significantly decreased, and resistance to DCFs increased in *MfSre1* knockdown transformants (Jiang *et al.*, 2019). In this study, the knockdown of *MfOfd1* did not influence the integrity of the cell wall in transformants. Under SDS stress, the inhibition rate of transformants was similar to the wild‐type isolate. Unlike *MfSre1*, knockdown of *MfOfd1* caused reduced tolerance to stress. The transformants were more sensitive to glycerol, d‐sorbitol, and NaCl than the wild‐type isolate. These results suggest that the *MfOfd1* gene might be involved in membrane integrity.

ROS are important signal transduction and defence substances produced by a plant active resistance reaction (Baxter *et al.*, 2013; Mühlenbock *et al*., 2007). Fungi need to activate their ROS detoxification and improve ROS tolerance for successful infection. Under oxidative stress, the inhibition of mycelial growth of the knockdown transformants was remarkably higher than that of the wild‐type isolate Bmpc7, suggesting that the *MfOfd1* gene is involved in the mycelial response to oxidative stress. Furthermore, the expression of *MfOfd1* was induced by treatment with H_2_O_2_, indicating that *MfOfd1* is indispensable for the fungal response to oxidative stress. The mechanisms of how *MfOfd1* regulates the response to oxidative stress should be investigated in the future.

Fungicide sensitivity assays showed that knockdown of *MfOfd1* did not affect the sensitivity of *M. fructicola* to DMI (propiconazole and tebuconazole) and SDHI (boscalid) fungicides. However, all the transformants were more sensitive to DCF (iprodione and dimethachlon) fungicides. DCFs are a class of fungicides that act on histidine and mitogen‐activated protein (MAP) kinase in the osmotic signal transduction pathway (Motoyama *et al.*, 2005; Yoshimi *et al.*, 2005; Luo *et al.*, 2012). The increased sensitivity of *MfOfd1* knockdown transformants to DCFs further proved that the *MfOfd1* gene plays an important role in the osmotic signal transduction pathway in *M. fructicola*. In our previous study it was found that the sensitivity to DCFs decreased in *MfSre1* knockdown transformants, indicating that *MfSre1* negatively regulates sensitivity to DCFs and osmotic stress (Jiang *et al.*, 2019). Therefore, it could be possible that both *MfOfd1* and *MfSre1* are involved in the response to osmotic stress in *M. fructicola*, but one is a positive regulator and the other is a negative regulator.

In conclusion, knockdown of the *MfOfd1* gene did not affect vegetative growth, but resulted in a decline in conidiation and affected the osmotic stress signal transduction pathway and tolerance to oxidative stress. At the early stage of infection, the ROS burst in plants strongly inhibits fungal development and the *MfOfd1* gene plays an important role in virulence through increasing the tolerance to the ROS burst. However, how *MfOfd1* regulates the downstream network and responds to the ROS burst is still not clear. The specific mechanism of *MfOfd1* in the infection process and osmotic stress signal transduction pathway of *M. fructicola* should be further studied.

## 4 EXPERIMENTAL PROCEDURES

### 4.1 Fungal isolate and growth conditions

The wild‐type single spore isolate Bmpc7 of *M. fructicola* was collected from a peach orchard in the United States and stored on filter paper at −20 °C (Luo *et al.*, 2008). Isolate was cultivated on PDA at 22 °C for 3 days in the dark. The fungal isolate was grown on 20% vegetable‐juice agar medium (V8, 200 ml V8 juice and 20 g agar per litre) for 2 weeks at 22 °C in the dark for sporulation (Lee *et al.*, 2010). For DNA and RNA extraction, 8–10 agar plugs containing mycelium were transferred to 40 ml potato dextrose broth and incubated at 22 °C on a 150 rpm orbital shaker for 36 hr in the dark. The genomic DNA was extracted using the EASYspin Plant Genomic DNA Extraction Kit (Aidlab Biotechnologies Co.).

### 4.2 RNA‐Seq, read quality, and data analysis

High‐throughput RNA‐Seq sequencing was used to detect the gene expression of 21 samples at seven stages (each stage with three technique replications) of 0, 1, 3, 6, 12, and 24 hpi, and spore germination (sg) stage during the infection of *M. fructicola* on peach fruits. In order to ensure the quality and reliability of data analysis, it is necessary to remove reads with adapters containing N (base information cannot be determined) and low quality (base number of Qphred ≤ 20 accounts for more than 50% of the entire read length) bases. In addition, the original data were checked for sequencing error rates and  guanine‐cytosine content distribution to obtain clean reads for subsequent analysis. Hierarchical Indexing for Spliced Alignment of Transcripts (HISAT) software (http://ccb.jhu.edu/software/hisat/index.shtml) was used to do genomic localization analysis of the filtered reads (Daehwan *et al.*, 2015).

According to the comparison results, the corresponding reads of each transcript were counted and standardized using the fragments per kilobase million (FPKM) method. Gene differential expression was analysed by DESeq2 software (http://www.bioconductor.org/packages/release/bioc/html/DESeq2.html). First, the read count was normalized (Anders and Huber, 2010), then the *p* value calculation model (negative binomial distribution) was used to calculate the probability of hypothesis testing and finally multiple hypothesis testing was corrected (calculation method:BH) to obtain the false discovery rate value. The differential gene screening standard was *p*_adj_ < .05.

### 4.3 Cloning and identification of *MfOfd1*

The transcriptome of *M. fructicola*‐infected peach fruits in the early stage (1 hpi) was analysed. It was found that the *MfOfd1* gene was significantly up‐regulated, suggesting that it is important for pathogenicity. The structure and amino acids sequence were predicted by Softberry (http://www.softberry.com/berry.phtml?topic=fgeneshandgroup=programsandsubgroup=gfind) software. To search its homologous proteins, a BLAST search of the predicted amino acid sequence was performed in the National Center for Biotechnology Information (NCBI) database using the protein BLASTP program. Phylogenetic analysis of Ofd1 proteins was performed with MEGA using amino acid sequences and a phylogenetic tree was generated by MEGA v. 7.0. The functional domains of MfOfd1 were predicted using the TMHMM Sever v. 2.0 (http://www.cbs.dtu.dk/services/TMHMM/) and InterProScan (https://www.ebi.ac.uk/interpro/search/sequence-search) programs.

The full length of the *MfOfd1* fragment was amplified by the primer pair Ofd1‐For and Ofd1‐Rev (Table S1). Identification of the exons and introns was determined by comparison of *MfOfd1* genomic DNA and cDNA sequences.

### 4.4 Vector construction and fungal transformation

Homologous recombination was combined with CRISPR/Cas9 to obtain the knockdown transformations (Figure S3). The upstream (982 bp) and downstream (595 bp) fragments of the *MfOfd1* gene in Bmpc7 and the fragment of hygromycin B resistance phosphotransferase gene (*HPH*, 1,414 bp) cassette in pSKH vector were amplified (Yun, 1998; Jiang *et al.*, 2019). Knockdown constructs were produced by double‐jointed PCR using three amplicons (upstream fragment, downstream fragment, and *HPH* cassette). The specific identification site of Esp3Ⅰ FastDigest (Thermo Scientific) was used to digest the pmCas9 empty vector at 37 °C for 15 min. The 20 bp before NGG (5′–3′) in the CDS region of *MfOfd1* was selected as the specific sequence of single‐guide RNA (sgRNA), and its specificity was confirmed through the local BLAST in the Bmpc7 genome. The sequence was synthesized in the form of forward primer and reverse primers (Table S1) and sticky ends (5′‐ACCT‐3′, 5′‐AAAC‐3′) were added at the 5′ ends (Liang *et al.*, 2018), then inserted into the digested pmCas9 vector by T4 DNA ligase (TaKaRa). The inserts in plasmids were sequenced to confirm their correctness.

To perform PEG‐mediated protoplast transformation, *M. fructicola* protoplasts were first prepared by digesting fresh mycelia with cell wall lyase (Lysing Enzymes from *Trichoderma harzianum*, Sigma) at 30 °C and 150 rpm for 4 hr (Jiang *et al* *.*, 2019). The digested suspension was centrifuged at 4 °C and 1,500 × g for 10 min, the supernatant was removed, and the precipitation was resuspended with STC (1.2 M sorbitol; 10 mM Tris‐HCl, pH 7.5; 50 mM CaCl_2_) solution. The fresh protoplasts suspension (200 μl, 10^8^/ml) was mixed gently with the transformation fragments (40 μl, >250 ng/μl) and pOfdCas9 vector (40 μl, 500 ng/μl), and placed on ice for 20 min. Then 1.2 ml PEG solution (60% PEG, mol. wt. 3,350; 10 mM Tris‐HCl, pH 7.5; 10 mM CaCl_2_) was added to the protoplast–DNA mixture and it was incubated at room temperature for 25 min. Then 1 ml STC solution was slowly added to the mixture and it was mixed well. The transformation mixture (450 μl) was spread onto a Petri dish (9 cm diameter) containing 20 ml regeneration agar medium (1 M sucrose, 0.1% yeast extract, 0.1% casein hydrolysate, 1.5% agar) at about 45 °C, and the dish was incubated in the dark at 22 °C. Once protoplast germination had been observed under the microscope, the regeneration agar medium was covered with water agar medium containing hygromycin B (150 μg/ml). The targeted gene knockdown transformants were selected on PDA with hygromycin B (200 μg/ml) for two generations and screened by PCR analysis for further confirmation.

### 4.5 DNA extraction and validation of knockdown transformants

The wild‐type isolate Bmpc7 and the knockdown transformants were cultured in PDB for 36 hr, and DNA was extracted using the EASYspin Plant Genomic DNA Extraction Kit (Aidlab Biotechnologies Co.). Integration of the hygromycin resistance gene in knockdown transformants was verified by the primer pair HF and HR, which amplified a 1,414 bp fragment. The primer pairs 5‐MfOfd1‐For and Check‐hyg‐Rev, and Check‐hyg‐For and 3‐MfOfd1‐Rev were used specifically to amplify the homologous fragment with partial fragments of left or right flanking regions, 1,748 and 1,046 bp, respectively. The primer pair MfOfd1‐C/Z‐For and MfOfd1‐C/Z‐Rev was used to identify whether transformants are homozygotes (1,829 bp) or heterozygotes (876 and 1,829 bp).

### 4.6 RNA extraction and RT‐qPCR

Total RNA was extracted using the EASYspin Plus Total RNA Extraction Kit (Aidlab Biotechnologies Co.). Genomic DNA was removed from total RNA by DNase (Thermo Fisher Scientific Inc.), and cDNA was synthesized by ReverAid First Strand cDNA Synthesis Kit (Thermo Scientific). The *β*‐*tubulin* gene was selected as the internal reference gene to determine the relative expression of target genes, and the fragment of 157 bp was amplified by primer pair NF and NR according to the cDNA sequence of *β*‐*tubulin* in Bmpc7 (Ma *et al.*, 2003; Schmittgen and Livak, 2008). The primer pairs for RT‐qPCR are given in Table S1. Expression of the *MfOfd1* gene was detected by RT‐qPCR with the primer pair MfF/MfR. RT‐qPCR was performed in a CFX96 Real‐Time PCR detection system (Bio‐Rad) using SYBR Green I fluorescent dye (Aidlab) in 20 μl volumes with 2 μl cDNA and 0.5 μl of each primer (10 μM). The experiments were performed with three independent biological repeats. The expression of the *MfOfd1* gene was normalized to the expression of the *β‐tubulin* gene, and relative gene expression was calculated with the comparative *C*_t_ (2^−ΔΔ^ *^C^* ^t^) method (Wong and Medrano, 2005).

### 4.7 Determination of mycelial growth, sporulation, and spore germination

All the wild‐type isolate and knockdown transformants were inoculated on PDA and MMA at 22 °C for 5 days to investigate the colony morphology and mycelial growth as described previously (Hu *et al.*, 2011). Spores were collected from colonies on 20% V8 agar for 2 weeks, and the number of conidia was counted under a microscope with a haemocytometer. To measure the spore size and evaluate the spore germination, spore suspension (100 μl, 3 × 10^4^/ml) was uniformly spread on water agar medium, 100 spores were randomly selected under the microscope, and the size of spores was measured perpendicularly. Spores germination was evaluated after incubation for 6 hr. These experiments were performed in three independent biological replications.

### 4.8 Virulence assay

The virulence assay was performed using the susceptible peach cultivar *Prunus persica* ‘Fei Cheng’. Holes (5 mm deep) were generated on the surface of fruits using a cork borer (5 mm diameter) and the holes were inoculated with mycelial plugs. All of the inoculated fruits were put into plastic boxes that were covered with cling film to maintain high humidity and incubated at 22 °C. Brown rot lesion size was measured at 24, 48, and 72 hr post‐inoculation. Three fruits were used for each strain in a treatment and the experiment was conducted three times.

### 4.9 Sensitivity assay to stress

To assess the integrity of the cell wall and cell membrane, PDA was amended with 600 μg/ml Congo Red or 0.01% SDS. For osmotic stress, PDA was amended with 150 g/L glycerol, 1.2 M d‐sorbitol, 0.6 M NaCl, or 0.7 M MgSO_4_. For oxidative stress, PDA and PDB were supplemented with 6 mM H_2_O_2_, and the expression level of *MfOfd1* was evaluated. The wild‐type isolate and the transformants were cultured on PDA in triplicate with the aforementioned stress conditions at 22 °C for 3 days.

### 4.10 Sensitivity to fungicides

The sensitivity to fungicides was investigated in the wild‐type isolate and transformants. For DCFs, sensitivity to iprodione and dimethachlon was assessed on fungicide‐amended PDA at 0.2 and 0.5 μg/ml, respectively. For DMIs, propiconazole and tebuconazole were added to PDA at 0.2 and 0.2 μg/ml, respectively (Yuan *et al.*, 2013). For SDHIs, boscalid was added to MMA at 0.8 μg/ml (Chen *et al.*, 2014). The wild‐type isolate and transformants were cultured in triplicate at 22 °C for 3 days. Colony diameters were measured and expressed as the percentage of growth inhibition.

### 4.11 Statistics

Multiple comparison was performed for the fitness data and statistical differences were evaluated by one‐way analysis of variance (ANOVA) with the least‐significant difference (LSD) test in SPSS v. 21.0 software at α = 0.05 (SPSS Inc.). For the difference of gene expression, fitness, and virulence between wild‐type isolate Bmpc7 and the knockdown transformants, the significance was determined by *t* test (***p< .001, **p < .01, *p < .05).

## CONFLICT OF INTEREST

The authors declare no conflict of interest.

## Data Availability

Transcriptomic data can be found in GenBank (https://www.ncbi.nlm.nih.gov/genbank/) with accession numbers SAMN12871599 to SAMN12871619. The sequence of MfOfd1 can be found in GenBank with accession no. MN515052.
