# Supplementary material for: MfOfd1 is crucial for stress responses and virulence in the peach brown rot fungus Monilinia fructicola
Source: Mol Plant Pathol. 2020 Apr 21;21(6):820–33. doi: 10.1111/mpp.12933 (PMC7214477; doi:10.1111/mpp.12933)
Supplement: Supplementary file 1 [file MPP-21-820-s001.doc]

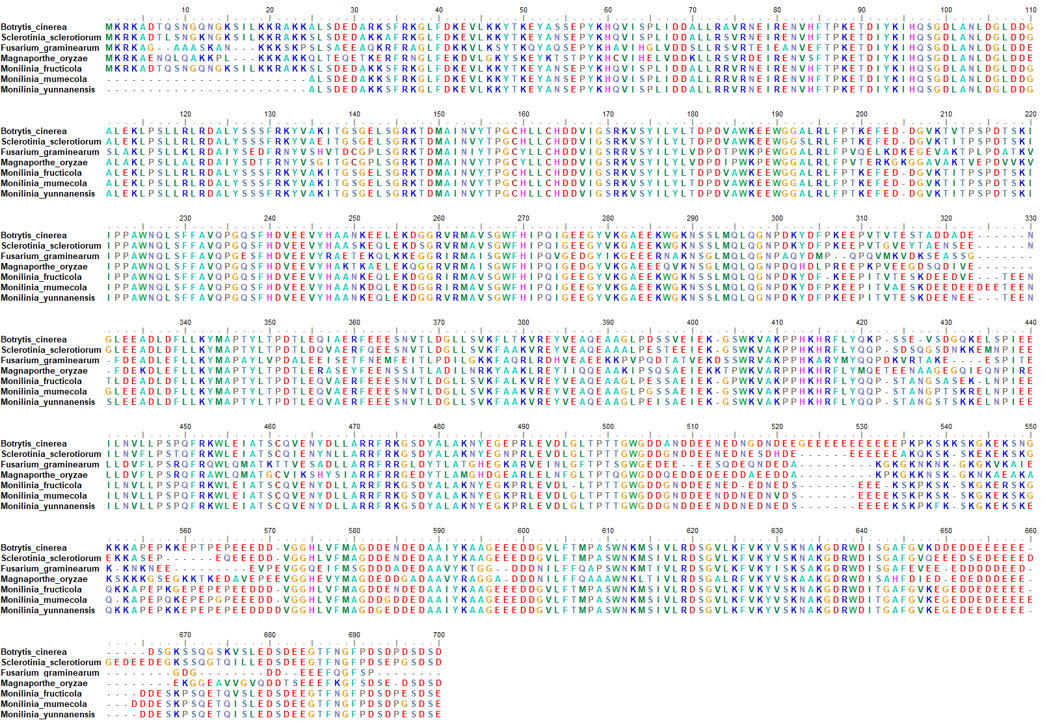


Fig. S1. Amino acid sequences of MfOfd1 aligned with Ofd1 proteins from *Botrytis cinerea* (XP_001558891.1), *Sclerotinia sclerotiorum* (XP_001597651.1), *Maganaporthe oryzae* (EHA53897.1), *Monilinia mumecola* and *Monilinia yunnanensis*.
