# Supplementary material for: MfOfd1 is crucial for stress responses and virulence in the peach brown rot fungus Monilinia fructicola
Source: Mol Plant Pathol. 2020 Apr 21;21(6):820–33. doi: 10.1111/mpp.12933 (PMC7214477; doi:10.1111/mpp.12933)
Supplement: Supplementary file 2 [file MPP-21-820-s002.doc]

Fig. S2. Schematic diagram of the *MfOfd1* gene replacement approach. The binding sites of the primers used for PCR analysis of knockout transformants are indicated. (A) The diagram of homologous fragments construction for transformation. (B) PCR analysis for knockout transformants.
