# Supplementary material for: MfOfd1 is crucial for stress responses and virulence in the peach brown rot fungus Monilinia fructicola
Source: Mol Plant Pathol. 2020 Apr 21;21(6):820–33. doi: 10.1111/mpp.12933 (PMC7214477; doi:10.1111/mpp.12933)
Supplement: Supplementary file 3 [file MPP-21-820-s003.doc]

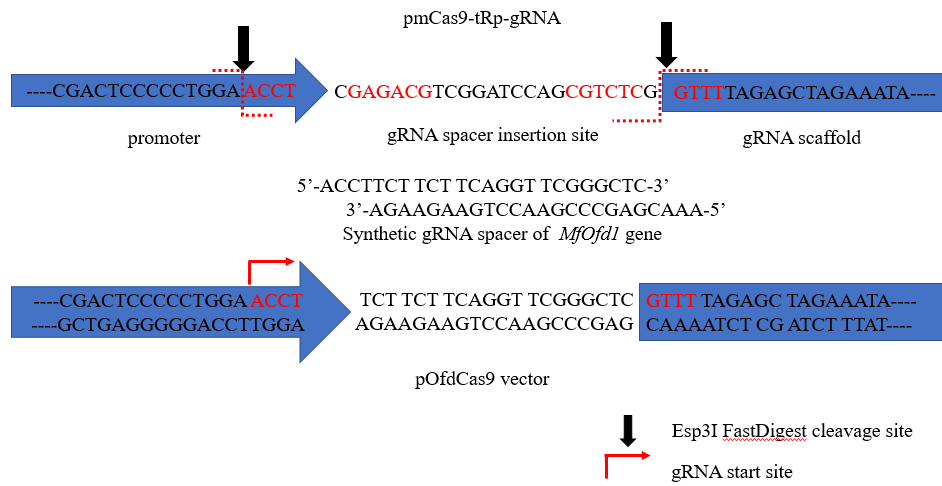


Fig. S3. Diagram of the modified target gene *MfOfd1* gRNA cassettes in pmCas9-tRp-gRNA vector. The sequence of red was recognized by Esp3Ⅰ FastDigest enzyme and cleavage. The gRNA spacers are synthesized by annealing the sense and antisense oligonucleotides with 5’-ACCT and 3’-CAAA overhangs and inserted into Esp3Ⅰ-digested pmCas9 empty vector by T4 DNA ligase, generated for pOfdCas9 vector.
