# Supplementary material for: MfOfd1 is crucial for stress responses and virulence in the peach brown rot fungus Monilinia fructicola
Source: Mol Plant Pathol. 2020 Apr 21;21(6):820–33. doi: 10.1111/mpp.12933 (PMC7214477; doi:10.1111/mpp.12933)
Supplement: Supplementary file 4 [file MPP-21-820-s004.doc]

Fig. S4. PCR verification of knockout transformants. (A) The primer pair 5-MfOfd1-For and Check-hyg-Rev was used specifically to amplify the homologous fragment with a partial fragment of left flanking region (1748 bp); (B) The primer pair Check-hyg-For and 3-MfOfd1-Rev was used to amplify the homologous fragment with a partial fragment of right flanking region (1046 bp); (C) The primer pair HF and HR was used to amplify the whole Hygromycin resistant gene (1414 bp); (D) The primer pair MfOfd1-C/Z-For and MfOfd1-C/Z-Rev was used to amplify the fragment containing the *MfOfd1* gene (876 bp and 1829 bp).
