# Supplementary material for: MfOfd1 is crucial for stress responses and virulence in the peach brown rot fungus Monilinia fructicola
Source: Mol Plant Pathol. 2020 Apr 21;21(6):820–33. doi: 10.1111/mpp.12933 (PMC7214477; doi:10.1111/mpp.12933)
Supplement: Supplementary file 5 [file MPP-21-820-s005.doc]

Table S1 Primers for *MfOfd1* gene clone, knockdown and verification

| Primer names | Sequence (5’—3’) |
| --- | --- |
| Ofd1-For | CAAGATGAAGCGTAAGGCAGATA |
| Ofd1-Rev | GGGCCTTGAAGGGTATACTATCTA |
| Ofd1-Nest-For | GGTGGAAGGATTAGAATGGCTG |
| M13F | TGT AAA ACG ACG GCC AGT |
| M13R | CAG GAA ACA GCT ATG ACC |
| pmCas9F | GCCTCTTCGCTATTACGCCA |
| pmCas9R | CTGAACCATCCTTTGACCAC |
| MfOfd1-CRISPR-For | ACCTTCTTCTTCAGGTTCGGGCTC |
| MfOfd1-CRISPR-Rev | AAACGAGCCCGAACCTGAAGAAGA |
| 5-MfOfd1-For | CGAGAAAACGTTCACTTCACAC |
| 5- MfOfd1-Rev | TCAATATCATCTTCTGTCGAGTGGTTTAGCAACTTTCCAAGG |
| 3-MfOfd1-For | AGATGCCGGATCCACTTAACATGGCAGGCGATGATGAGAA |
| 3- MfOfd1-Rev | TCTGATAAACGAATGCGAAATG |
| 5-Nest- MfOfd1-For | ATGTGATTGGTAGCCGCAAAGT |
| 3-Nest- MfOfd1-Rev | ATGCCGATACACCATTTCCATC |
| HF | TCGACAGAAGATGATATTGAAGGAG |
| HR | GTTAAGTGGATCCGGCATCT |
| Up-Nest-R | TCGCCTCGCTCCAGTCAATG |
| Down-Nest-F | AGGGCGAAGAATCTCGTGCTTT |
| Check-hyg-For | AGGAATCGGTCAATACACTACAT |
| Check-hyg-Rev | ATGTAGTGTATTGACCGATTCCT |
| MfOfd-C/Z-F | TTGCCGAACGTTTTGAGGAG |
| MfOfd-C/Z-R | CGTCGTCCTCTTCCTCTTCATC |
| MfF | GGATGGGGCGATGATGGTAATG |
| MfR | GGCTCTCCTTTGGGCTCTGGT |
| NF | AACCTTGAAGCTCAGCAACC |
| NR | GAAATGGAGACGTGGGAATG |
